# Hybrid Modelling by Machine Learning Corrections of Analytical Model Predictions towards High-Fidelity Simulation Solutions

**DOI:** 10.3390/ma14081883

**Published:** 2021-04-10

**Authors:** Frederic E. Bock, Sören Keller, Norbert Huber, Benjamin Klusemann

**Affiliations:** 1 Institute of Materials Mechanics, Helmholtz-Zentrum Hereon, 21502 Geesthacht, Germany; soeren.keller@hereon.de (S.K.); norbert.huber@hereon.de (N.H.); benjamin.klusemann@leuphana.de (B.K.); 2 Institute of Product and Process Innovation, Leuphana University of Lüneburg, 21335 Lüneburg, Germany

**Keywords:** machine learning, analytical model, finite element model, artificial neural networks, model correction, feature engineering, physics based, data driven, laser shock peening, residual stresses

## Abstract

Within the fields of materials mechanics, the consideration of physical laws in machine learning predictions besides the use of data can enable low prediction errors and robustness as opposed to predictions only based on data. On the one hand, exclusive utilization of fundamental physical relationships might show significant deviations in their predictions compared to reality, due to simplifications and assumptions. On the other hand, using only data and neglecting well-established physical laws can create the need for unreasonably large data sets that are required to exhibit low bias and are usually expensive to collect. However, fundamental but simplified physics in combination with a corrective model that compensates for possible deviations, e.g., to experimental data, can lead to physics-based predictions with low prediction errors, also despite scarce data. In this article, it is demonstrated that a hybrid model approach consisting of a physics-based model that is corrected via an artificial neural network represents an efficient prediction tool as opposed to a purely data-driven model. In particular, a semi-analytical model serves as an efficient low-fidelity model with noticeable prediction errors outside its calibration domain. An artificial neural network is used to correct the semi-analytical solution towards a desired reference solution provided by high-fidelity finite element simulations, while the efficiency of the semi-analytical model is maintained and the applicability range enhanced. We utilize residual stresses that are induced by laser shock peening as a use-case example. In addition, it is shown that non-unique relationships between model inputs and outputs lead to high prediction errors and the identification of salient input features via dimensionality analysis is highly beneficial to achieve low prediction errors. In a generalization task, predictions are also outside the process parameter space of the training region while remaining in the trained range of corrections. The corrective model predictions show substantially smaller errors than purely data-driven model predictions, which illustrates one of the benefits of the hybrid modelling approach. Ultimately, when the amount of samples in the data set is reduced, the generalization of the physics-related corrective model outperforms the purely data-driven model, which also demonstrates efficient applicability of the proposed hybrid modelling approach to problems where data is scarce.

## 1. Introduction

There is currently a surge in the application of machine learning algorithms in various fields of materials mechanics. In general, scientific and industrial research groups focus on the identification and utilization of one or more relationships along the process–structure–property–performance (p-s-p-p) chain [1]. In this domain, the application of machine learning techniques can be a key enabler for accelerated identification, characterization, understanding and optimization of processes, materials and parameters [2]. For instance, unique material descriptors can be qualified and quantified for material characterization [3,4,5]. Optimization and rapid design of novel manufacturing methods and involved materials [6,7] can be achieved, and inaccurate measurement techniques can be corrected [8]. The generation of knowledge and understanding to enable improved predictions of mechanical properties and performances, among others, can be acquired on the basis of experimental and/or numerical data in combination with machine learning models [9,10]. Furthermore, the integration of well-established physical laws into data-driven machine-learning models can be very beneficial to perform highly accurate predictions and inferences of involved phenomena [11,12]. However, besides these physics-informed machine learning methodologies, Chinesta et al. [13] introduced a hybrid modelling approach, where an efficient physics-based model shows some prediction errors that are corrected by a subsequent data-driven model to ultimately reach the anticipated solution.

Deployment of only either data-driven predictive models or calibrated physics-based models is accompanied with respective disadvantages based on each approach. Calibration of physics-based models can be difficult, expensive and time-costly even for domain experts, as it can be challenging or even impossible for physical quantities of interest to be accessible through experimental measurements. It is almost unattainable to represent the reality via such models only through data assimilation [14]. For purely data-driven approaches, the relevant relationships between input and output variables are required to be satisfactorily represented in the data set, as there is an absence of internal physics-related variables [15]. This creates the demand for a comprehensive database for the learning algorithm to represent those relationships. For problems that are still largely unknown, this can be a suitable approach; however, when some relations are already known, it is inefficient to create the need of a big-data-set for ensuring it represents all relevant aspects of the underlying physical laws that are required to be learned “from scratch” by the machine-learning algorithm [16]. In a study by Liu et al. (2020) [17], a data-driven surrogate model to predict the plane-strain stress intensity factor at the crack tip during fracture toughness tests is built with an adaptability and efficiency that is comparable to an analytical or empirical solution within their physical problem domains. In [17], high-fidelity numerical simulations are used to create the data-base for correlation of dimensionless inputs and outputs. However, due to the purely data driven approach, a vast number of computationally expensive simulation solutions are required for sufficient training of the surrogate model, which could create challenges for accuracy and generalization when switching to an experimental data source for training. Purely data-driven approaches can be beneficial for those problems where few relationships are identified, as they can help to detect hidden relationships in data; however, when established physical-laws apply and available data is scarse or biased, the utilization of physically-related data-driven approaches can be countervailing and utile [18,19].

Consequently, studies are focused on the aim to represent physical problems and their associated behaviour through physics-based models as well as on the pursuit to account for the deviation between those models and the reality via data-driven corrections. González et al. (2019) [20] performed corrections for hyperelastic models based on data-driven machine learning, whereas Ibáñez et al. (2018) [21] implemented a hybrid approach consisting of constitutive modelling and data-driven machine learning correction of plasticity models. In a manufacturing application example for metal forming production, Havinga et al. (2020) [22] performed real-time predictions via a hybrid modelling approach that contains physics-based simulations those predictive deviations to the real process are eliminated via an additional corrective model. Overall, the specific employment of machine learning models alongside governing physics-based relationships allows for highly valid predictions within materials mechanics and its related fields.

Generally, physics-based models might show prediction errors but as these deviations are systematic and not owed to noise, they can be accounted for separately. In combination, physics-based models and deviation models can be used to correctly predict a real system’s behaviour. The advantages of using a calibrated model based on well-established physics, even when it shows deviations to reality, are that the compensating corrective model applied for achieving high prediction accuracy requires fewer samples and less complexity to approximate the deviation, since it is usually considerably less non-linear than the problem itself. This opens up the possibility to easily correct a physics-based model with a relatively simple correction model towards true/desired data points to assure an adequate representation of the behaviour by the system of interest [13]. Chupakhin et al. [8] introduced a corrective artificial neural network (ANN) for the hole drilling method, where residual stresses are determined based on measurements of elastic material behaviour, which are corrected towards the solution of a plasticity-including finite element (FE) model by an ANN. Thus, as opposed to correcting numerical models by empirical observations, in this case, biased experimental measurements can be successfully corrected through an ANN driven by physics-based numerical data.

The objective of this study is to build a hybrid model, consisting of a physics-based model and a data-driven corrective model, with low prediction errors even when training data is scarce. A semi-analytical model, originally proposed by Hu et al. [23], is employed as low-fidelity physics-based model, including a number of simplifications and a subsequent ANN is used to correct this solution towards a true reference solution provided by an FE model considered as high-fidelity. As example use-case, laser shock peening (LSP)-induced residual stress distributions over the specimen depth in aluminium alloy AA2024 are considered. In particular, since the representation of the relationships between residual stress distributions in dependence of LSP-generated pressure pulses over time is severely simplified in its semi-analytical model solution, we aim for the complementary corrective approach. Ultimately, high-fidelity approximation of the desired system behaviour is achieved by combining semi-analytical and ANN-correction models, which are both computationally efficient. In addition, when the data used for training, validation and testing is reduced, the predictions obtained via this hybrid modelling approach exhibit less errors than a purely data driven model. We propose a hybrid process model consisting of data-driven correction-learning of an LSP process model, which also shows good generalization ability, even when the parameter space of the training region is expanded and the available data becomes scarce.

## 2. Methods and Materials

The implemented corrective approach combines a semi-analytical model, which exhibits significant deviations in predictions outside its calibration parameter space, with a data-driven machine learning model correcting those deviations towards the solution of the high-fidelity model. The corrective model is required to be less complex, for solely representing a corrective component, compared to a purely data-driven prediction model mapping the more complex and complete relationships that are relevant. Additionally, this hybrid approach shows good generalization ability and also exhibits low prediction errors in an expanded input parameter space outside the parameter space used for training, as opposed to decreased generalization ability of a purely data-driven model, which is not physics-related. For the selected use-case of LSP, the residual stress distributions intended to be corrected are calculated via the semi-analytical model from Hu et al. [23]. An FE model was used for computing the desired reference residual stress distributions, which represent the true/desired data in this work. The correction task is developed through training, validating and testing of an ANN. Both numerical and semi-analytical models will be briefly introduced in the following two sections. For more details, the reader is referred to the original publications, as the focus of this study lies on the correction task where those models are assumed as black-box models and their detailed mechanisms are deliberately not intended to be relevant for the current study. (Note: the selected use-case LSP serves only as selected example. Generally speaking, the analytical model could be replaced by any physics-based model and the data from the FE model represents the corresponding, typically scarce, experimental data). Material parameters correspond to the aluminium alloy AA2024 in T3 heat treatment condition, frequently used in the aircraft industry for fuselage structures [24].

### 2.1. Laser Shock Peening

One of the main goals of the transportation industry is to reach weight, fuel and CO${}_{2}$ savings as well as increase the sustainability of engineering components [25]. For improving the fatigue life of light-weight materials such as aluminium alloys, LSP has gained attention in scientific research and industrial application developments. LSP is known as residual stress modification technique to introduce high and deep compressive residual stresses in metallic components [26]. These compressive residual stresses can be used to enhance fatigue properties of metallic structures, which is of high interest for damage tolerant design concepts, as applied in aircraft structures. However, compressive residual stresses are always accompanied by fatigue-critical tensile residual stresses due to stress equilibrium. During LSP, short-time (nanosecond regime), high-energy (Joule regime) laser pulses are used to convert material at the surface into plasma. Plasma expansion initiates mechanical shock waves that cause local plastic strains in the material. After relaxation of the dynamic process, a characteristic residual stress field is developed, which contains both: Relatively high compressive residual stresses and balancing tensile residual stresses. Experimental process observation is very challenging and requires great effort due to the magnitudes of physical quantities, such as plasma pressure as well as temperature, and the short time scale. The knowledge of the residual stress fields is essential for efficient application of LSP, motivating the development of suitable prediction tools. Modelling of the LSP process is challenging due to the short time scale of the process, which, so far, leads to imprecise experimental determination of physical quantities occuring during shock wave propagation and plasma formation, such as material strain rates up to $10^{6}\;s^{-1}$, plasma pressure of several GPa or the high plasma temperature; therefore, the utilized material model can exhibit determination inaccuracies regarding these quantities. There are various approaches to simulate the LSP process, such as FE models [27,28,29] or (semi) analytical models. While FE models represent the most commonly used modelling approach, to represent the three-dimensional physics involved in the complex LSP process, the considered semi-analytical model by Hu et al. [23] is computationally very efficient but does not provide any information on tensile stresses because stress equilibrium is neglected.

Other simplifications include the assumption of an infinite instead of finite specimen thickness as well as single value calculations of stresses at distinct model locations as opposed to averaged stress calculation based on extrapolation of finite element integration points towards nodes, among others. Since the considered LSP system and FE model uses quadratic pulse spots, see Keller et al. [29], the underlying assumption of a circular spot in the semi-analytical model represents a further simplification in the current case.

Ultimately, the proposed correction approach is employed to achieve low prediction errors while simultaneously using the implied physics and maintaining the computational efficiency of the analytical model. Such a hybrid modelling approach is new in the context of LSP, where the number of publications on the application of machine learning approaches for the LSP process is scarce, overall. Frija et al. [30] optimized the LSP surface conditions by using an FE model exposed to the laser-induced pressure pulse as well as Design of Experiments (DoE) to infer related laser parameters. They extended the work by the use of an ANN to efficiently predict significant characteristics of numerical compressive residual stress profiles and approximated a simplified 1st-order linear slope of residual stresses [31]. In this study, it is aimed for efficiently predicting the original non-linear distribution of compressive and tensile residual stresses, provided by an FE model, throughout the complete depth of the specimen. Wu et al. [32] also performed predictions of LSP-induced residual stresses via an ANN based on the laser profile and laser energy purely based on experimental data; thereby, not explicitly considering relevant physical relationships. Mathew et al. [33] used an ANN for the prediction and optimization of residual stress distributions induced by LSP, where the relative importance of four process parameters on residual stresses is investigated purely based on experimental data. In this work, the proposed hybrid model generates highly accurate predictions that are physics-related via the corrective approach of a physics-based analytical model.

### 2.2. Physical Models

In the following, the pressure pulse input definition for both physical models as well as the semi-analytical model and high-fidelity FE model, are described.

#### 2.2.1. Pressure Pulse Definition for Physical Models

**Figure 1:**
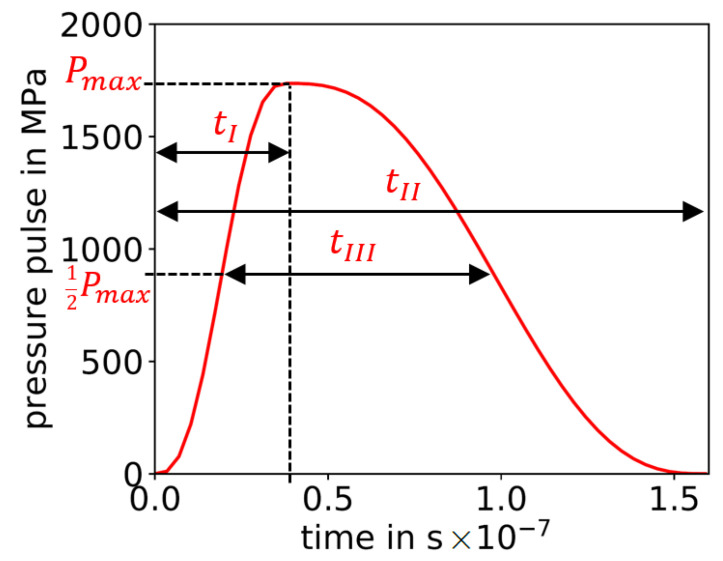
Pressure pulse over time including its uniquely defining parameters: Maximum pressure $P_{max}$, time of maximum pressure $t_{I}$ and pulse duration $t_{II}$. As additional information, the full width at half maximum is given by $t_{III}$.

**Figure 2:**
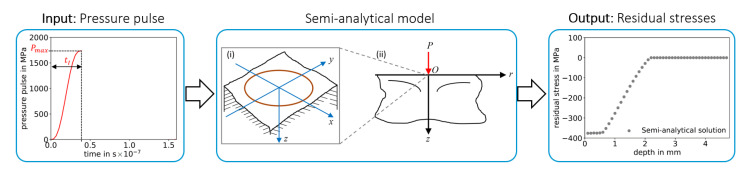
Illustration of the semi-analytical model by Hu et al. [23] for computing residual stresses induced by pressure pulse from Figure 1. Circular pressure pulse area (i) (in red) on the half-space model, which is simplified in (ii) as a concentrated normal load (in red) in the axisymmetric half-space model. Figures (i) and (ii) are republished with permission of the American Society of Mechanical Engineers ASME from [23].

**Figure 3:**
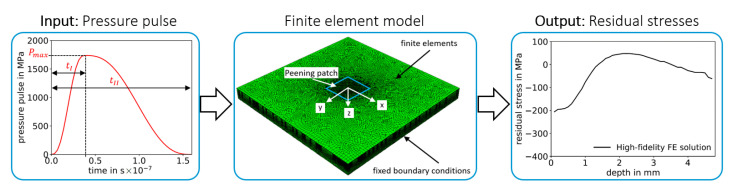
Finite element process model for computing residual stresses induced by pressure pulse from Figure 1.

The definition of the pressure pulses over time, in Figure 1, is utilized as input for the semi-analytical model, see Figure 2a, and for the high-fidelity FE model, see Figure 3a. The pressure pulse over time is uniquely defined in this work based on three pressure pulse parameters: Maximum pressure $P_{max}$, the time of maximum pressure $t_{I}$ and the pulse duration $t_{II}$, see Figure 1. This pressure pulse function is preferred in the utilized ABAQUS solver of the FE analysis since it is differentiable and assures efficiency and stability of the FE solver [34]. Note that the original semi-analytical model by Hu et al. [23] is slightly modified by using this pressure pulse as input, instead of laser parameters. Note: The pulse duration $t_{II}$ is not considered in the semi-analytical model, as described in the following Section 2.2.2.

#### 2.2.2. Low-Fidelity Model — Semi-Analytical Model

A semi-analytical LSP process model to predict residual stress profiles depending on the plasma pressure is developed by Hu et al. [23], which is adopted in this study. In the process model, a semi-infinite space and rotational symmetry are assumed since a circular laser focus is considered. Furthermore, single laser pulse impacts are modelled instead of a laser pulse sequence. The residual stress profile is evaluated along the symmetry axis. The LSP process of a single laser pulse impact is split into two phases: Loading and relaxation. During the loading phase, the pulse pressure from t=0 to $t_{I}$ is considered as input and during the relaxation phase, the resulting residual stresses are calculated (note that the pressure pulse interval from $t_{I}$ to $t_{II}$ is not considered in this model). Plasma induced stresses that are present during the loading phase are assumed to be superposed and fully developed stress fields that are caused by time dependent surface tractions of the plasma pressure, representing the elastic solution. The stress field caused by a single traction is described by closed-form expressions corresponding to the equation found for single forces, see Timoshenko and Goodier [35]. Plastic material deformation and resulting stresses are calculated by the McDowell Hybrid Algorithm [36]. A strain-rate dependent material model, including isotropic and kinematic hardening is employed. The strain-rate dependency of the yield stress is modelled by the Johnson–Cook model, where material parameters are listed in Table 1. After the application of the plasma pressure, the residual stress field is calculated during the relaxation phase; therefore, stresses are incrementally reduced while plastic deformation is taken into account to match stress and strain boundary conditions of an axisymmetric half space. A stress equilibrium is not calculated by this algorithm, as opposed to the FE analysis, which is explained in the following Section 2.2.3. For more details on the semi-analytical model, the interested reader is referred to the original work by Hu et al. [23]. Overall, the main involved physical phenomena are considered in the semi-analytical model but to a substantially simplified extent leading to a relatively narrow parameter space, where in combination with a subsequent correction, the desired high fidelity solution of the FE model within a much wider parameter space can be reached, nevertheless.

#### 2.2.3. High-Fidelity Model — FE Model

The FE LSP-process model, set up to calculate residual stresses in AA2198 [29] and adopted to AA2024 [37] in the author’s previous works, is used in this work to generate a database with the plasma pressure as input and residual stress profiles as output, see Figure 3. The LSP process model consists of a cuboid with dimensions of 60 mm × 60 mm × 4.8 mm and the depth is discretized with an element size of 0.02 mm next to the surface. Sides parallel to *x-z* and *y-z* plane are modelled with fixed boundary conditions, whereas sides parallel to *x-y* plane are considered as free surfaces. The plasma pressure caused by a single laser pulse is modelled as a time dependent surface traction that is uniformly distributed within the peened area. The temporal pressure profile is varied to set up the data set for training, validation and testing. A square of 3 × 3 laser pulses is simulated without overlap, where the square focus size is 3 mm × 3 mm. Residual stresses below the centred laser pulse are averaged layer-wise to calculate a residual-stress-over-depth profile, which has shown to be valid by comparison to experiments [29,37]. The LSP process model consists of approximately $1.4\times 10^{6}$ continuum elements with reduced integration (C3D8R). The Johnson–Cook material model [38] is utilized, where the used material parameters for AA2024 are summarized in Table 1 for convenience. Nine pressure pulses are simulated in Abaqus/Explicit. A relaxation time of 50 μs is simulated between each pulse, which ensures that the dynamic process reaches a state sufficiently close to equilibrium to prevent significant interaction between two consecutive laser pulses, modelled as pressure loadings. After the simulation of all laser pulses, a final quasi-static implicit simulation (Abaqus/Standard) is conducted to determine the residual stress equilibrium. For further details on the model, the interested reader is referred to [29].

**Table 1:** Elastic and Johnson–Cook material parameter representative for aluminium alloy AA2024 in T3 heat treatment condition with an equivalent plastic strain rate ${\dot{\varepsilon }}_{P,0}=2\times 10^{-4}\;s^{-1}$ according to [39].

Parameter	Symbol	Unit	Value
Density	ρ	g/cm${}^{3}$	2.8
Young’s modulus	*E*	GPa	74
Poisson’s ratio	ν	–	0.33
Quasi-static yield strength	*A*	MPa	350
Strengthening coefficient	*B*	MPa	972
Strain hardening exponent	*n*	–	0.73
Dynamic strain hardening coefficient	*C*	–	0.01

### 2.3. Artificial Neural Networks

**Figure 4:**
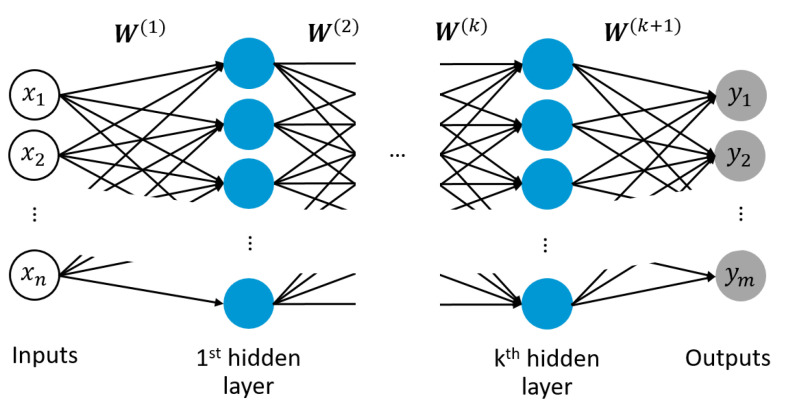
Schematic of a multi-layered neural network with input layer, *k* hidden layer and output layer, including weight vectors W of edge connections between neurons of adjacent layers for correlating *n* number of inputs $\left[x_{1},x_{2},\ldots ,x_{n}\right]$ to *m* number of outputs $\left[y_{1},y_{2},\ldots ,y_{m}\right]$.

An ANN represents a computational instrument that can “learn” to correctly map an input to an output via the adjustment of weights. The initial idea of the perceptron was to mimic the behaviour of a neuronal cell in the nervous system of the human brain [40]. Feed forward neural networks are multiple perceptrons composing one or more layers of neurons, where each neuron computes an output based on inputs from the previous layer and an inherent non-linear activation function. The signal is processed in an unidirectional forward direction from input to output throughout the network, where the input signal is progressively transformed into an output signal, see Figure 4. ANNs can be trained to approximate any non-linear relationship [41]. Training of such networks is achieved through back propagating error minimization via gradient descent. The error resulting from the difference between current network output and true/desired output (which is known in a supervised learning task) is minimized by adapting the behaviour of individual neurons through adjusting the weights of the connecting edges between those neurons. The learning rate defines the step size per weight update during gradient descent. For the implementation of an adaptive learning rate, different learning rate optimizers are available, such as Adam [42], Momentum [43] or Adagrad [44], among others. Ultimately, the network represents a mapping rule that is based on provided training examples and is only valid for the space contained in those samples; thus, these networks are not suitable for extrapolating predictions outside the training sample domain. A brief description of a feed forward neural network with back propagating error minimization is provided in the following.

Overall, achieving sufficient training and validation of an ANN depends on the amount of available data, network complexity and the nonlinear nature of the particular relationships to be approximated. To obtain a good ability of the ANN to generalize well, the prediction error on training and validation data sets need to be both low and similar [45], as it indicates that neither underfitting nor overfitting has occurred during training. To prevent overfitting on the training data, learning can be terminated based on the “early stopping” criterion, which is fulfilled as soon as the prediction performance on the validation data set (outside the training data set) is no longer improved during training, even though the error on the training set is still decreasing.

## 3. Methodology

First, patterns are generated with pressure pulses and residual stresses from both semi-analytical and FE models. The resulting pairs of semi-analytically and numerically determined residual stress profiles compose the training data set for the corrective task of the ANN. Second, the ANN is trained, validated and tested. Third, the ANN is utilized for correcting semi-analytical residual stress profiles generated by an expanded pulse parameter range that was not contained in the previously utilized training, validation and test data sets. This methodology is described in detail in the following.

**Figure 5:**
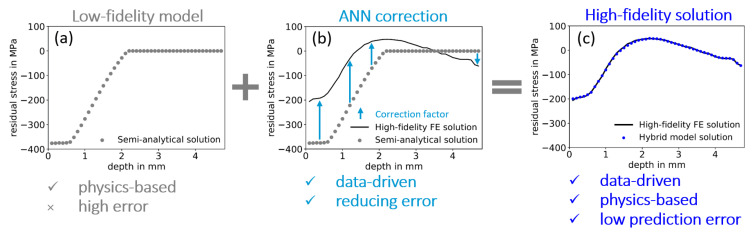
Schematic of hybrid model implementation for prediction of laser shock peening (LSP)-induced residual stresses: (**a**) Residual stresses predicted by the semi-analytical model exhibiting relatively high prediction errors compared to the high fidelity FE solution which is compensated by (**b**) a correction factor “learned” by an artificial neural network (ANN), leading to (**c**) the validated high-fidelity prediction with low errors, i.e., the hybrid model solution.

As illustrated in Figure 5, the corrected predictions for LSP-induced residual stresses contain the estimates from the physics-based semi-analytical model and a corrective term from the corrective ANN that accounts for the deviation between semi-analytical stresses and numerical stresses to generate the desired high-fidelity solution.

### 3.1. Data Preparation

For correcting the coherent residual stress profiles, stress values are discretized over the depth in the form $d\left(i\right)/d_{max}$, leading to 47 points from d(0.1) to d(4.7), as surface stresses are disregarded (specimen thickness = 4.8 mm). Included in the input is the information of the known pressure pulses used to generate each residual stress profile with the semi-analytical model. The maximum pressure $P_{max}$ of the particular pulse serves as normalization for all stress values of the respective profile. Since residual stress profiles can converge towards zero, a division by zero or very small stress values during normalization is prevented by a uniform shift of all residual stress values above zero by adding twice the material’s yield strength (note that the quasi-static yield strength *A* is used, here) denoted with $\sigma_{y}$, see Equations (1) and (2). To enable the prediction of correction factors that produce results of high accuracy, one point of the depth discretization is considered at a time. Thus, the depth at which the correction factor for the residual stresses shall be determined is used as the final input. This yields the following dimensionless input space, including shifted and scaled residual stresses over depth:

(1) $$X^{i}:=\left\{\frac{\sigma_{ana,1}^{i}+2\sigma_{y}}{P_{max}^{i}},\frac{\sigma_{ana,2}^{i}+2\sigma_{y}}{P_{max}^{i}},\ldots ,\frac{\sigma_{ana,47}^{i}+2\sigma_{y}}{P_{max}^{i}},\frac{j}{47}\right\}$$

with *i* as the sample number, *j* as the discretization step of the depth in the range from 0.1 mm to 4.7 mm and ${P^{i}}_{max}$ as the maximum pressure of the specific sample. The dimensionless output is the correction factor and defined as:

(2) $$Y^{i}:=\left\{\frac{\sigma_{ana,j}^{i}+2\sigma_{y}}{\sigma_{FE,j}^{i}+2\sigma_{y}}\right\}$$

with $\sigma_{ana,j}^{i}$ and $\sigma_{FE,j}^{i}$ being the residual stresses at the depth j/47, computed by semi-analytical model and finite element model, respectively. Using a single output, where each output corresponds to a different depth j/47, one can observe a smooth curve as a result for the complete continuous distribution since the ANN is forced to smoothly approximate this depth dependency. A smooth curve of the output is obtained when the input is scanned with j/47 through the depth. With each *j* value, a corresponding stress correction at the output is received. The use of physically normed inputs and outputs allows for making predictions in a much wider process parameter range than that used for training of the ANN [46], which can be highly beneficial.

A total number of 82 numerical and semi-analytical sample pairs with pressure pulse parameter ranges listed in Table 2, have been utilized. With the proposed depth discretization of 47, this led to a total of 82 × 47 = 3854 patterns that composed the complete data set. The data is randomly split into training, validation and test data sets with an 80/10/10 ratio with the constraint of a stratified $P_{max}$ value range into eight classes, i.e., equidistant subintervals from 800 MPa to 2200 MPa. Thereby, each class is represented in the respective data sets to ensure the ranges of maximum pulse values are similar in training, validation and test data sets, respectively. Ultimately, training, validation and test data sets consisted of 3102, 376 and 376 patterns, respectively. Scaling of inputs and outputs was executed to remain in value ranges of [−1,1] and [1,5], respectively. The corrected residual stresses are obtained by solving Equation (2) with respect to absolute values $\sigma_{ana,j}^{i}$.

### 3.2. Hyperparameters of ANN

The ANN consists of two hidden layers each containing 30 neurons, respectively. The sigmoid function is utilized as the activation function of each layer, except for the final layer, where a linear activation function is implemented to obtain continuous values in the proposed regression task. Gradient descent during mean squared error (MSE)-loss optimization through weight adjustments is enhanced with an adaptive learning rate according to the Adam optimizer. Furthermore, early stopping is implemented to enable training without any overfitting, as training is stopped as soon as the generalization error, i.e., MSE-loss on validation data set, is not decreased any further. Before early stopping is executed, a patience of 1000 further epochs is used to assure that no local minimum on the validation set MSE within this consecutive 1000-epoch-range leads to the stopping. The workflow of this study, consisting of data pre-processing, ANN development and result analysis, has been executed with the open-source libraries Scikit-learn and Keras in conjunction with JupyterNotebook frontend and Tensorflow back-end.

## 4. Development and Evaluation of ANN-Correction Model

The ANN correction model proposed here is developed and evaluated in two steps. First, the input feature space only contains semi-analytical residual stresses distributed over depth, normalized with the maximum of the corresponding pulse pressure, where the correction predictions still exhibit significant errors. Second, the input feature space is enriched with additional salient features according to a consistent dimensionality analysis, which led to a decrease of those prediction errors. The prediction performances are evaluated with two metrics: Determination coefficient ($R^{2}$) and mean squared error (*MSE*). $R^{2}$ is defined as

(3) $$R^{2}=1-\frac{\sum_{i=1}^{N}{\left(y_{i}-y_{i,pred}\right)}^{2}}{\sum_{i=1}^{N}{\left(y_{i}-y_{mean}\right)}^{2}}\;,$$

where $y_{i}$ represents the true value, $y_{i,pred}$ the predicted value, $y_{mean}$ the mean of the true values and *N* the number of sample values. *MSE* is defined as

(4) $$MSE=\frac{1}{N}\sum_{i=1}^{N}{\left(y_{i}-y_{i,pred}\right)}^{2}.$$

### 4.1. Approach 1: Consideration of Only Semi-Analytical Residual Stresses as Input

**Figure 6:**
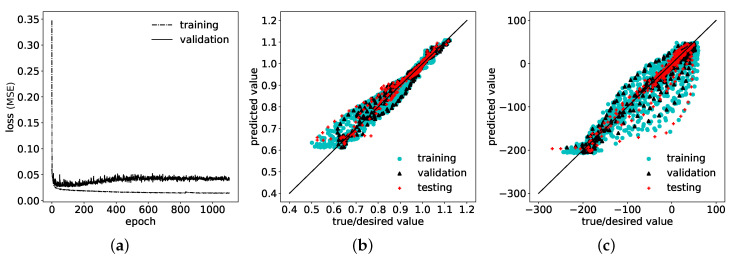
(**a**) Learning curves: Mean squared error (MSE)-loss function values minimized via weight adjustment of the ANN on training set and simultaneous MSE for predictions on validation set with training-set weights over number of epochs during training. (**b**) Determination coefficient $R^{2}$ for correction factor (ANN output) achieved by ANN on training, validation and test data sets. (**c**) Determination coefficient $R^{2}$ for related residual stresses attained by ANN on training, validation and test data sets.

**Figure 7:**
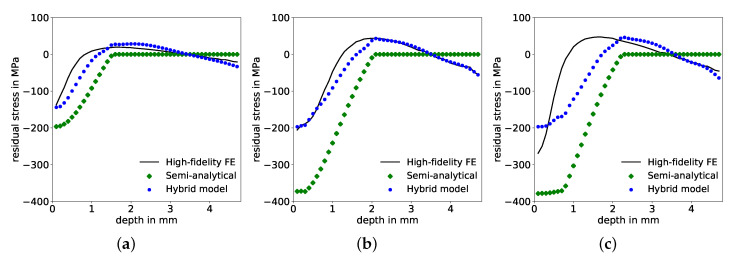
Comparison of residual stress distributions over depth predicted by the FE model, semi-analytical model and hybrid model for three exemplary test samples with pulse parameters maximum pressure $P_{max}$, time of maximum pressure $t_{I}$ and pulse duration $t_{II}$ of (**a**) 1236 MPa, 15.1 ns, 85 ns; (**b**) 1639 MPa, 37.7 ns, 145 ns; and (**c**) 1820 MPa, 13 ns, 65.7 ns.

In this first approach, the input for the corrective ANN prediction consists only of the semi-analytically determined residual stresses, normalized with the maximum pressure value of the pulse, Equation (1). The so-called “learning curves”, i.e., values of the loss function (the MSE) on training and validation data sets during training (over the number of epochs), shown in Figure 6a, indicate a significantly lower MSE for predictions on the training data than on the validation data. In other words, the network has been over-fitted to the training data and shows low ability to generalize well, as the prediction error is increased on data points outside the training data set. Correspondingly, the $R^{2}$ values for the correction factor, presented in Figure 6b, and the resulting residual stresses, shown in Figure 6c, exhibit deviations between true/desired values and predicted values. Specifically, $R^{2}$ values for the correction factor, Equation (2), reached 97.08%, 96.65% and 94.94% on training, validation and test set, respectively, see Table 3. For the predictions of corrected residual stresses, these deviations are even greater, with $R^{2}$ values of 91.14%, 91.35% and 81.88% for training, validation, and test sets, respectively, see Table 3. Comparisons of input, output and corrected residual stresses of three exemplary test samples are shown in Figure 7, where the corrections of the semi-analytical stresses are not in good agreement with the desired FE solutions. The error of the stress predictions is decreased through the correction but not to a satisfactory extend. In order to improve corrective model predictions with respect to an increased determination coefficient $R^{2}$ and a decreased MSE, additional information needs to be provided in the input space for the ANN.

**Figure 8:**
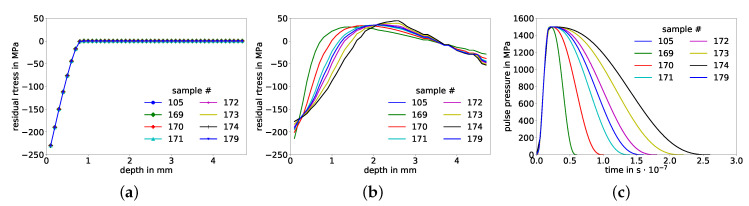
(**a**) Super-imposed but indistinguishable residual stress distributions over depth predicted by the semi-analytical model for different pressure pulses, i.e., identical inputs for the corrective ANN-model. (**b**) Corresponding output targets: Eight unique residual stress distributions over depth predicted by the FE model and (**c**) corresponding distinctive pressure pulses over time that were used as input for both models, exhibiting different pulse durations but identical maximum pressures and times of respective maximum pressures.

As mentioned in Section 2.2.1, the pulse duration $t_{II}$ is not considered in the semi-analytical model according to its input definition, the pressure pulse duration is only considered until $t_{I}$. As a result, samples whose corresponding pressure pulses differ uniquely only in duration will cause predictions of identical residual stress distributions, see Figure 8. Mathematically, this is a non-injective relationship, inadequate to be represented by any function, i.e., the same input could certainly not be correlated to multiple different outputs via the ANN-model of this first approach, where only residual stresses over depth serve as input. Consequently, as pulse duration $t_{II}$ affects the prediction result, it needs to be considered in the input space for the corrective model.

### 4.2. Approach 2: Adding Salient Features to the Input Space

In order to enable a unique mapping between inputs and outputs, additional input features are identified via a dimensionality analysis and are added to the input space. In accordance with the Buckingham Π theorem [47], a required minimum number of dimensionless parameters can be defined to sufficiently describe the physical problem. Thus, besides the analytical stresses $\sigma_{ana}$ and maximum pressure $P_{max}$, the pressure pulse time quantities $t_{I}$, $t_{II}$ and $t_{III}$ are included. To connect those temporal measures to mechanical properties *E* and ρ, the wave speed $c=\sqrt{E/\rho }$ is also considered. Ultimately, the peened area $A_{peened}$ is used to complete the set of five dimensionless quantities:

(5) $$\Pi_{1}=\frac{\sigma_{ana}}{P_{max}},\;\Pi_{2}=\frac{t_{I}}{t_{II}},\;\Pi_{3}=\frac{t_{III}}{t_{II}},\;\Pi_{4}=t_{III}\sqrt{\frac{E}{\rho \cdot A_{peen}}},\;\Pi_{5}=\frac{P_{max}}{E}.$$

Adding dimensionless information that is based on a consistent dimensionality analysis to the input space leads to a reduction of inaccuracies, which is in agreement with a study based on a similar input definition for an ANN [46]. Subsequently, this leads to a further reduction of prediction’s MSE and increase of R${}^{2}$ compared to the first approach presented in Section 4.1. All input-output pairs can be uniquely identified by the ANN. Accordingly, the modified input is described with:

(6) $$X^{i}:=\left\{\frac{\sigma_{ana,1}^{i}+2\sigma_{y}}{P_{max}^{i}},\frac{\sigma_{ana,2}^{i}+2\sigma_{y}}{P_{max}^{i}},\ldots ,\frac{\sigma_{ana,47}^{i}+2\sigma_{y}}{P_{max}^{i}},\frac{{t_{I}}^{i}}{{t_{II}}^{i}},\frac{{t_{III}}^{i}}{{t_{II}}^{i}},{t_{III}}^{i}\sqrt{\frac{E}{\rho \cdot A_{peen}}},\frac{P_{max}^{i}}{E},\frac{j}{47}\right\}.$$

**Figure 9:**
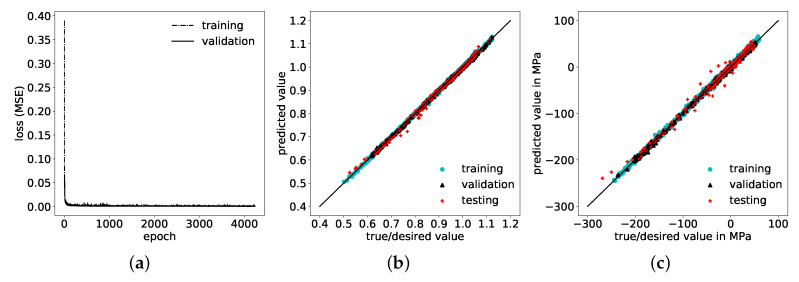
(**a**) Learning curves: MSE-loss function values on training and validation data sets over number of epochs during training and (**b**) corresponding prediction values of the correction factor versus true values, and of (**c**) the corresponding residual stresses.

**Figure 10:**
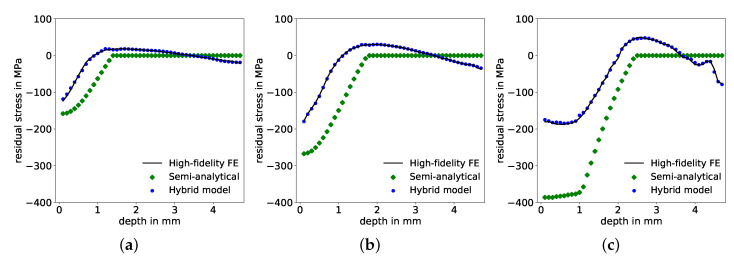
Comparison of residual stress distributions over depth predicted by the FE model, semi-analytical model and hybrid model for three test samples with maximum pressure $P_{max}$, time of maximum pressure $t_{I}$ and pulse duration $t_{II}$ of (**a**) 1144 MPa, 38.9 ns, 137 ns; (**b**) 1390 MPa, 22.2 ns, 140 ns; and (**c**) 2039 MPa, 49.5 ns, 243 ns, respectively.

This dimensionless formulation ensures that all dependencies are scaled without loss of generality. In comparison to the first approach, the previous bias and variance indicated in the learning curves in Figure 6a is reduced, as the final MSE-loss is further reduced on both training as well as on validation data sets, respectively, and both converged towards similar values, see Figure 9a. Hence, prediction results improved significantly on all three data sets, with respect to increased determination coefficients $R^{2}$ each above 99% for the correction factors, see Figure 9b and also for the corrected residual stresses, see Figure 9c. The MSE on the test set declined simultaneously to a maximum of $3.9\times 10^{-5}$ and 28.63 MPa${}^{2}$ for correction factors and corrected residual stresses, respectively, see Table 4. There is good agreement between the corrected prediction and the desired values of the residual stresses throughout the complete depth, as demonstrated by three examples from the test data set in Figure 10.

## 5. Generalization of Hybrid Model

**Figure 11:**
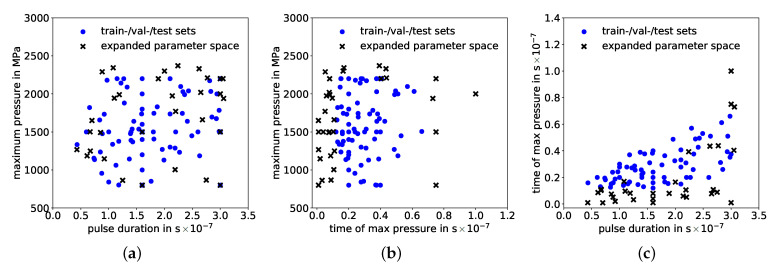
Sample positioning in the expanded parameter space: Maximum pressure over (**a**) pulse duration and over (**b**) time of maximum pressure as well as (**c**) time of maximum pressure over pulse duration.

An evaluation of the generalization ability is performed by expanding the input parameter space, i.e. value ranges of pressure pulse parameters: Maximum pressure $P_{max}$, time of maximum pressure $t_{I}$ and pulse duration $t_{II}$, to respective ranges that were not used for training, validation and testing, as shown in Figure 11 and Table 5. The lower bound of the maximum pressure range remained at 800 MPa because there is an almost insignificant contribution to residual stress formation by pressure pulses with a maximum below 800 MPa. In addition, extension of maximum pressures above 2400 MPa becomes physically unfeasible. Ultimately, there is no significant expansion but only minor exceedances for $P_{max}$ values beyond the training space. Lower bounds of pulse durations were decreased from 12 ns to 1 ns and upper bounds increased from 66 ns to 100 ns. The expanded-space data-set contained 35 samples. With this expanded parameter space, deviations between semi-analytical and high-fidelity solutions can be adequately corrected by the ANN and its trained range of correction factors.

The “learned” range for the correction factors is [0.5090,1.1189]; thus, the deviation between analytical and numerical model has to be correctable by values within that range in order to achieve the anticipated solutions. Restrictions are inevitable when the required factor for an appropriate correction lies outside this range. In this case, no correction is performed by the ANN and the analytical input is also the output. This corresponds to setting the correction factor to 1.0. Thus, the default prediction, in a worst-case scenario, is the provided input—the prediction of the semi-analytical model, which can be noticed clearly and used as an indicator for no correction having been performed. Essentially, an extrapolating prediction on an expanded parameter space can only be performed as long as the output of the ANN, i.e., the required correction factor, still lies in the value range of the training data set.

### 5.1. Setup of Purely Data-Driven ANN as Benchmark

The prediction performance of the hybrid model is benchmarked against the estimations of a purely data-driven ANN trained directly with pressure-pulse-over-time as input and residual-stresses-over-depth provided by the FE-model as output, without the consideration of any physics-based model. In the following, this purely data-driven ANN is briefly explained. Essentially, no corrective task is performed and the input consists of 47 discretized pressure values and the respective terms defined in the dimensionality analysis with

(7) $$X_{direct}^{i}:=\left\{\frac{P_{1}^{i}}{{P^{i}}_{max}},\frac{P_{2}^{i}}{{P^{i}}_{max}},\ldots ,\frac{P_{47}^{i}}{{P^{i}}_{max}},\frac{{t_{I}}^{i}}{{t_{II}}^{i}},\frac{{t_{III}}^{i}}{{t_{II}}^{i}},{t_{III}}^{i}\sqrt{\frac{E}{\rho \cdot A_{peen}}},\frac{{P^{i}}_{max}}{\sigma_{y}},\frac{j}{47}\right\}.$$

The output space contains the residual stress values, where constant discretization over specimen depth of the residual stresses is used, similar to the output discretization of the output space for the corrective model by

(8) $$Y_{direct}^{i}:=\left\{\frac{\sigma_{FE,j}^{i}+2\sigma_{y}}{\sigma_{y}}\right\}$$

where superscript *i* refers to the sample number and subscript *j* to the depth discretization step of 0.1 mm in the range from 0.1 mm to 4.7 mm.

The previous ANN architecture consisting of two hidden layers with respective 30 neuron and sigmoid activation functions is used to avoid any artificial influence of the ANN architecture in the benchmark. Likewise, early stopping is implemented to avoid overfitting during training. Normalization of inputs to [−1,1] and outputs to [1,5] is performed, as for the hybrid model.

### 5.2. Comparison of Physics-Based Hybrid Model and Purely Data-Driven ANN

**Figure 12:**
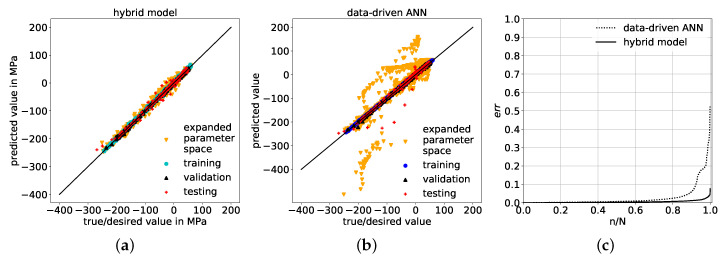
Juxtaposition of predicted values and true/desired values on training, validation, test sets and expanded parameter space data set, achieved by (**a**) the physics-based hybrid model and (**b**) the purely data-driven ANN, respectively. (**c**) shows the relative error of samples n normalized with the total number of samples N, sorted from low to high err values on the data set with expanded parameter space generated by hybrid model and data-driven ANN.

With a comparison of the physics-based corrective prediction in Figure 12a to a purely data-driven ANN prediction model in Figure 12b, an example for the benefits of a corrective physics-based ANN model over a purely data-driven ANN is provided. As can be seen in the comparison of the predictions in the expanded parameter space, predictions that are purely based on data exhibit pronounced errors, which is not the case for the ANN where physical laws are considered in the contained analytical model solution. This good prediction performance is a consequence of remaining within the trained range of correction factors as well as a result of the enhanced prediction ability of the hybrid model itself, which is owed to the decreased complexity of the correction problem. Even though the R${}^{2}$ values for the data-driven ANN are both above 99% on training and validation sets as well as above 95% on the testing set, the MSEs on the expanded space are almost two orders of magnitude higher than the one of the physics-based corrective model and amounts to over 1700 MPa${}^{2}$, see Table 6. The MSE of the corrective model measures just below 31 MPa${}^{2}$. The determination coefficient $R^{2}$ of the corrective approach on the extrapolation data set is highly alike to the $R^{2}$ values on the other data sets and is still as high as 99.39%, whereas for the data-driven approach, the $R^{2}$ values are all above 99% on training and validation and above 95% on test data sets but drops down to 65% for predictions on the expanded parameter space.

The absolute value of the relative error of both physics-based corrective model as well as purely data-driven model is defined as err, according to [48], and computed via:

(9) $$err:=\left|\frac{d-y^{N}}{d}\right|$$

with true values *d*, predicted values *y* and number of samples *N*. The maximum err from the data-driven model is approximately 53% and just below 8% for the corrective model at n/N=1, as shown in Figure 12c, where the normalized number of samples is sorted from small to large err values. As a result, consideration of the problem’s physics through the semi-analytical model leads to a better generalization compared to using a purely data-driven predictor relying on the relevant physics to be represented (only) in the training data. In particular, via the corrective ANN, interpolation within its trained value range of correction factors can still be performed, even on the expanded parameter space; whereas via the data-driven ANN, extrapolating predictions are performed within the expanded parameter space, which is unfeasible for an ANN because its predictive function is fitted to the training data and becomes unreliable in a variable space for which no training data is available. So, in this use-case example, based on a physically reasonable extension of the parameter space, a physics-based correction model exhibits superior prediction performance over a data-driven model, under the condition that results can be adjusted with the trained range of correction factors to achieve the anticipated solution.

### 5.3. Data Reduction Effects on Hybrid Model and Data-Driven ANN Predictions

In this section, the prediction performances of the hybrid model and the data-driven ANN are juxtaposed while the total number of samples is reduced. The total data set is split into training, validation and test data sets via a constant data-split ratio of 80/10/10, throughout a reduction of the total data set from 100% to 20% by increments of 10%. Thus, a 100% data set consists of 66 training, 8 validation and 8 test samples (as in all previous sections); whereas a 20% data set contains 13 training, 1 validation and 1 test sample(s). The specific samples and total sample number in the expanded-space data set remained constant at 35. For each data-reduction step, the data split is performed randomly and three times, each time with a different random state, in order to avoid prediction results that depend on specific samples contained in the respective data sets. Consequently, the MSE average and standard deviation of the corresponding three prediction models are calculated and used for further evaluation.

**Figure 13:**
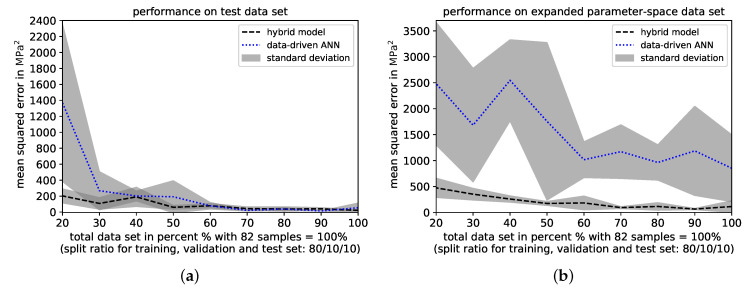
Comparison of prediction performances of hybrid model and direct ANN with respect to the average mean squared error (MSE) and standard deviation achieved on (**a**) the test data set and (**b**) the extrapolation data set, while reducing the amount of the total data set (training, validation and test data sets) from 100% to 20% in increments of 10%-steps, respectively. All MSE average values and standard deviations are based on three different MSEs and their respective standard deviations that are achieved on dissimilar data splits implemented by changing pseudo-random-states.

The hybrid model outperforms the data-driven ANN on the test data set with respect to an overall decreased mean MSE and continuously lower standard deviations. On average, the mean MSE is lower and its standard deviation decreased, when performing predictions with the hybrid model compared to the data-driven ANN. As shown in Figure 13a, these outperformances appear clearly once the amount of samples in the total data set in reduced below 60%, i.e., below a sample number of 49, as well as at the smallest total data set of 20%, respectively. On the extrapolation data set, the superior prediction ability of the hybrid model over the data-driven ANN is magnified with respect to a significantly lower average mean MSE and a substantially decreased standard deviation, see Figure 13b.

These outperformances could be due to several reasons. Primarily, the corrective ANN with its correction factor prediction is assumed to be more simple in complexity and in non-linearity than the residual stress prediction of the data-driven ANN. Consequently, the corrective ANN in combination with the semi-analytical model is more stable and robust in its predictions once the amount of data is reduced, in comparison to the data-driven ANN. In addition, there appears to be a higher dependence on specific samples being contained in the training and validation data sets for the data-driven ANN since the variation of mean MSE and standard deviation are more significant within an identical amount of data (but different random data splits). Ultimately, the proposed corrective approach, i.e., hybrid model consisting of the semi-analytical model and the corrective ANN, exhibits a number of benefits over a purely data-driven ANN, even more when the amount of data is scare or very limited, such as in DoE data sets.

## 6. Conclusions

In this study, a physics-based semi-analytical model, representing a rather simple but very efficient model, has been successfully combined with a corrective ANN into an hybrid prediction model to enhance its applicability range. Ultimately, low prediction errors were reached with respect to the desired high-fidelity solution, provided by a numerical FE simulation in the investigated use case of LSP. The high-fidelity numerical data could easily be replaced by experimental data, enabling correction towards empirical measurements. A number of prerequisites for adequately performing the correction task have been identified. Primarily, unique relationships between inputs and outputs need to exist, where redundancies in the data can be an indicator for non-unique relationships. These non-unique relationships may be compensated by using additional (salient) features identified via a consistent dimensionality analysis. Upon detectable uniqueness between inputs and outputs, low prediction errors are enabled. Essential findings for achieving a low prediction error in our specific problem domain are:

- Through the proposed corrective approach of a semi-analytical model, the solution of a high-fidelity numerical simulation is reached very efficiently.
- In particular, trained range of correction factors allows for a maximum adjustments of semi-analytical stresses of up to approximately 50% towards the desired high-fidelity solution.
- Generalized predictions for extended process parameter ranges can be achieved under the condition of correction factor values remaining within the training value range.
- Within the value range of trained correction factors, the generalization of the physics-based corrective approach within an expanded-parameter-space performs with significantly lower prediction errors compared to a purely data-driven generalization.
- When reducing the amount of available data during training, validation and testing, the generalization via the corrective approach demonstrated significantly reduced prediction errors compared to the purely data-driven model on both test set and expanded parameter-space data set, illustrating its ability to handle sparse data.

## Figures and Tables

**Table 2:** Pressure pulse parameter ranges of maximum pressure $P_{max}$, time of maximum pressure $t_{I}$ and pulse duration $t_{II}$ for training, validation and test data sets.

	$P_{\max}$ [MPa]	$t_{I}$ [ns]	$t_{\mathrm{II}}$ [ns]
Min.	800	12	43
Max.	2200	66	300

**Table 3:** Prediction metrics of trained ANN via Approach 1: $R^{2}$ (determination coefficient) and *MSE* (mean squared error) for correction coefficients as well as for corresponding residual stresses on training, validation and test data sets, respectively.

	Correction Factor		Residual Stresses	
Data Set	$R^{2}$ in %	MSE	$R^{2}$ in %	MSE in MPa${}^{2}$
Training	97.08	0.000466	91.14	399.21
Validation	96.65	0.000602	91.35	452.26
Test	94.94	0.000669	81.88	607.42

**Table 4:** Prediction metrics of the trained ANN via Approach 2: Determination coefficient $R^{2}$ and *MSE* for correction coefficients as well as corresponding residual stresses achieved on training, validation and test data sets, respectively.

	Correction Factor		Residual Stresses	
Data Set	$R^{2}$ in %	MSE	$R^{2}$ in %	MSE in MPa${}^{2}$
Training	99.95	$7\times 10^{-6}$	99.90	4.33
Validation	99.93	$12\times 10^{-6}$	99.86	7.38
Test	99.71	$39\times 10^{-6}$	99.15	28.63

**Table 5:** Expanded pressure pulse parameter ranges of maximum pressure $P_{max}$, time of maximum pressure $t_{I}$ and pulse duration $t_{II}$ as extrapolated parameter space in comparison to the ranges in the data set used for training, validation and testing, see Table 2.

		$P_{\max}$ in MPa	$t_{I}$ in ns	$t_{\mathrm{II}}$ in ns
Training, validation, test	Min.	800	12	43
	Max.	2200	66	300
Expanded parameter space	Min.	800	1	43
	Max.	2400	100	306

**Table 6:** Prediction metrics of the hybrid model and purely data-driven ANN: $R^{2}$ and *MSE* for residual stresses of samples in training, validation, test and expanded parameter space data sets.

	Hybrid Model		Data-Driven ANN	
Data Set	$R^{2}$ in %	MSE	$R^{2}$ in %	MSE in MPa${}^{2}$
Training	99.90	4.33	99.86	6.32
Validation	99.86	7.38	99.76	12.39
Test	99.15	28.63	95.89	137.58
Expanded space	99.39	30.17	65.00	1717.18

## Data Availability

The data presented in this study are available upon reasonable request from the corresponding author.
